# Transcriptomics-driven drug repositioning for the treatment of diabetic foot ulcer

**DOI:** 10.1038/s41598-023-37120-1

**Published:** 2023-06-20

**Authors:** Wirawan Adikusuma, Zainul Amiruddin Zakaria, Lalu Muhammad Irham, Baiq Leny Nopitasari, Anna Pradiningsih, Firdayani Firdayani, Abdi Wira Septama, Rockie Chong

**Affiliations:** 1 Borneo Research on Algesia, Inflammation, and Neurodegeneration (BRAIN) Group, Department of Biomedical Sciences, Faculty of Medicines and Health Sciences, University Malaysia Sabah, Jalan UMS, 88400 Kota Kinabalu, Sabah Malaysia; 2 Departement of Pharmacy, University of Muhammadiyah Mataram, Mataram, Indonesia; 3 Research Center for Vaccine and Drugs, National Research and Innovation Agency (BRIN), South Tangerang, Indonesia; 4 Faculty of Pharmacy, Universitas Ahmad Dahlan, Yogyakarta, Indonesia; 5 Research Center for Pharmaceutical Ingredients and Traditional Medicine, National Research and Innovation Agency (BRIN), South Tangerang, Indonesia; 6 Department of Chemistry and Biochemistry, University of California, Los Angeles, USA

**Keywords:** Computational biology and bioinformatics, Drug discovery, Genetics

## Abstract

Diabetic foot ulcers (DFUs) are a common complication of diabetes and can lead to severe disability and even amputation. Despite advances in treatment, there is currently no cure for DFUs and available drugs for treatment are limited. This study aimed to identify new candidate drugs and repurpose existing drugs to treat DFUs based on transcriptomics analysis. A total of 31 differentially expressed genes (DEGs) were identified and used to prioritize the biological risk genes for DFUs. Further investigation using the database DGIdb revealed 12 druggable target genes among 50 biological DFU risk genes, corresponding to 31 drugs. Interestingly, we highlighted that two drugs (urokinase and lidocaine) are under clinical investigation for DFU and 29 drugs are potential candidates to be repurposed for DFU therapy. The top 5 potential biomarkers for DFU from our findings are *IL6ST, CXCL9, IL1R1, CXCR2,* and *IL10*. This study highlights *IL1R1* as a highly promising biomarker for DFU due to its high systemic score in functional annotations, that can be targeted with an existing drug, Anakinra. Our study proposed that the integration of transcriptomic and bioinformatic-based approaches has the potential to drive drug repurposing for DFUs. Further research will further examine the mechanisms by which targeting *IL1R1* can be used to treat DFU.

## Introduction

Diabetes mellitus (DM) is a common chronic condition affecting 451 million people globally. By 2045, it is predicted that the number of DM patients would increase to 693 million^1^. Treatment for DM thus far has only been able to regulate blood sugar levels; unfortunately, it does not totally cure the condition. Improper management of DM can result in chronic problems; 15–25% of DM patients will experience diabetic foot ulcer (DFU), and 50–70% of relapses will occur within five years^2,3^. Amputation, death, and hospitalization rates are frequently high because of the refractory wound of DFUs^4^. DFU occurs in 85% of diabetic amputees before developing significant gangrene or infection^5^. Early detection is necessary to avoid long-term consequences. Hospitalization, disability, and mortality in DM patients will decrease if the risk of DFU can be identified early. Biomarkers are crucial for early clinical diagnosis, illness prevention, and disease progression prediction. In addition, finding and developing novel therapeutic medications or assessing therapeutic approaches is also essential^6,7^.

Genomic and biomedical information in the form of databases has rapidly become available, thanks to technological advances in experimental and computational biology. These comprise transcriptome data (such as human patient gene expression profiles, animal models of human diseases, small molecule treatments, etc.), other molecular profiling technologies, and publicly available databases, providing a previously unheard-of chance to enhance rational drug design in combination with the implementation of the network idea of drug targets and the power of phenotypic screening^8,9^. Transcriptome data can enable the identification and prioritization of biomarkers that can be used as potential therapeutic candidates^7^. In the past, several studies successfully identified promising results for various indications, such as inflammatory bowel disease^10^, dermatomyositis^11^, cancer^12–14^, and preterm birth^15^, by applying a transcriptomics-based computational drug repositioning pipeline. Consequently, we postulate that transcriptomic data can also help identify biomarkers for DFU and potential drug candidates for this disease.

**Figure 1:**
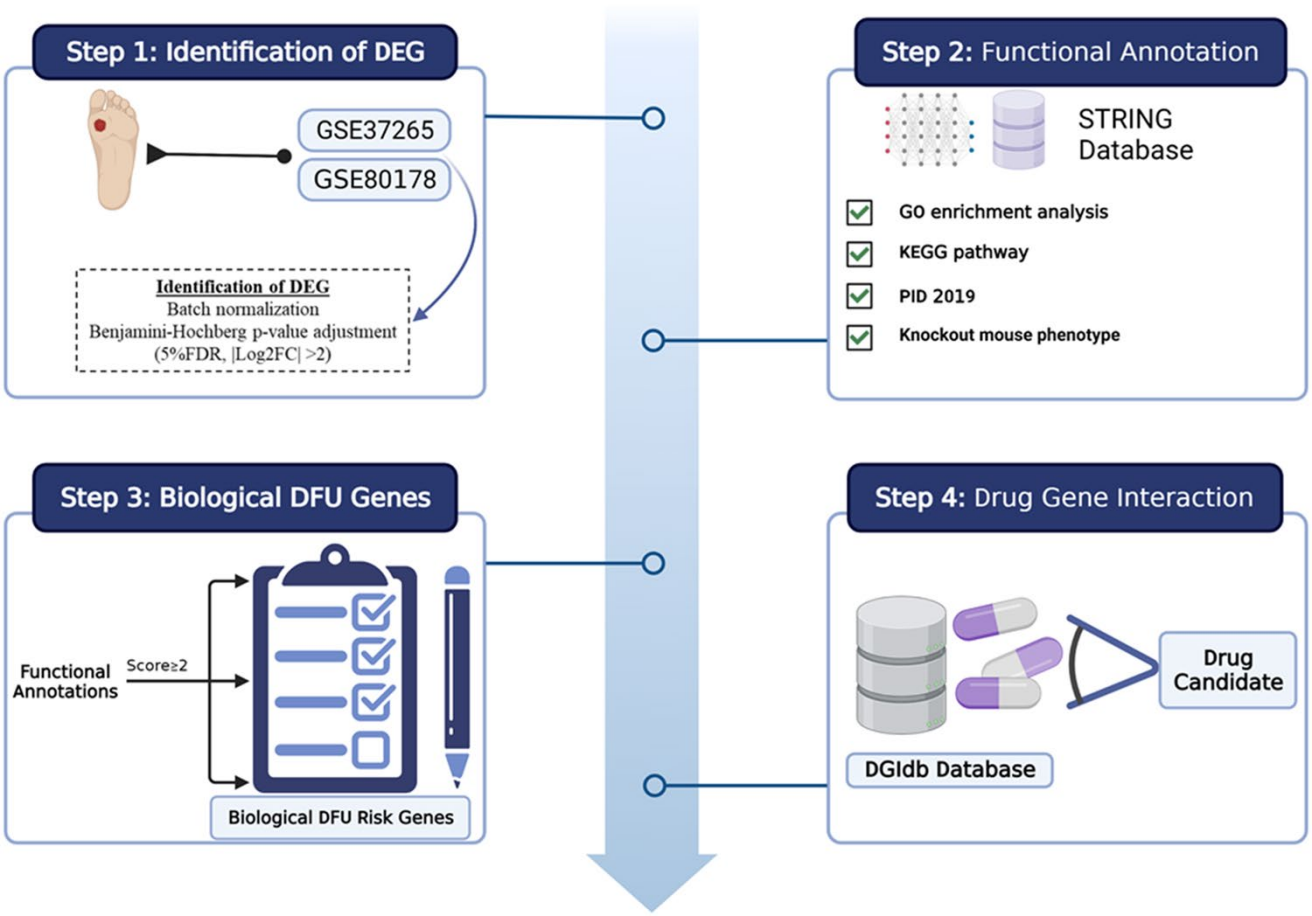
A workflow of the bioinformatics-driven drug repositioning pipeline for diabetic foot ulcer (DFU). This figure was created with BioRender.com under agreement number "QL24UF44NA".

Integrating transcriptomics and bioinformatics data could provide new insights into DFU pathogenesis by developing common transcription features. Here, we applied a transcriptomics-based computational drug repositioning pipeline to identify potential candidate therapy for DFU. This study was designed to discover the common genes among DFU patients. Detailed information on the study workflow is depicted in Fig. 1. The common DEGs and their roles in DFU were examined. Additionally, biological DFU risk genes were identified based on six functional annotations. As intended, higher scores that are given to candidate genes by the annotation process represent a more prominent biological influence that we termed “biological risk genes” for the pathogenesis of DFU. Subsequently, we used an *in-silico* workflow for the analyses from multiple databases to find prospective drugs or biomarkers for DFU. This information will be an important tool to not only drive biomarker discovery but can also drive drug repurposing for DFUs.

## Results

### Identification of differentially expressed genes for DFU

This study leveraged the previously published study datasets GSE37265 and GSE80178.

The GSE37265 and GSE80178 expression profile were identified using the R limma package to determine the differentially expressed genes **(**DEGs). We chose these two datasets with the following rationale. Firstly, we ensured that all the subjects included in our studies were human. Secondly, the sample type was healthy or diseased tissue from patients rather than a specific cell type, which was consistent with our goal for drug repositioning. Thirdly, we ensured that the datasets included in our study had complete data for analysis and had obtained ethical approval. Finally, we included diseased or normal skin samples and a control group without systemic or autoimmune diseases or relevant family history as inclusion criteria for our study.

**Figure 2:**
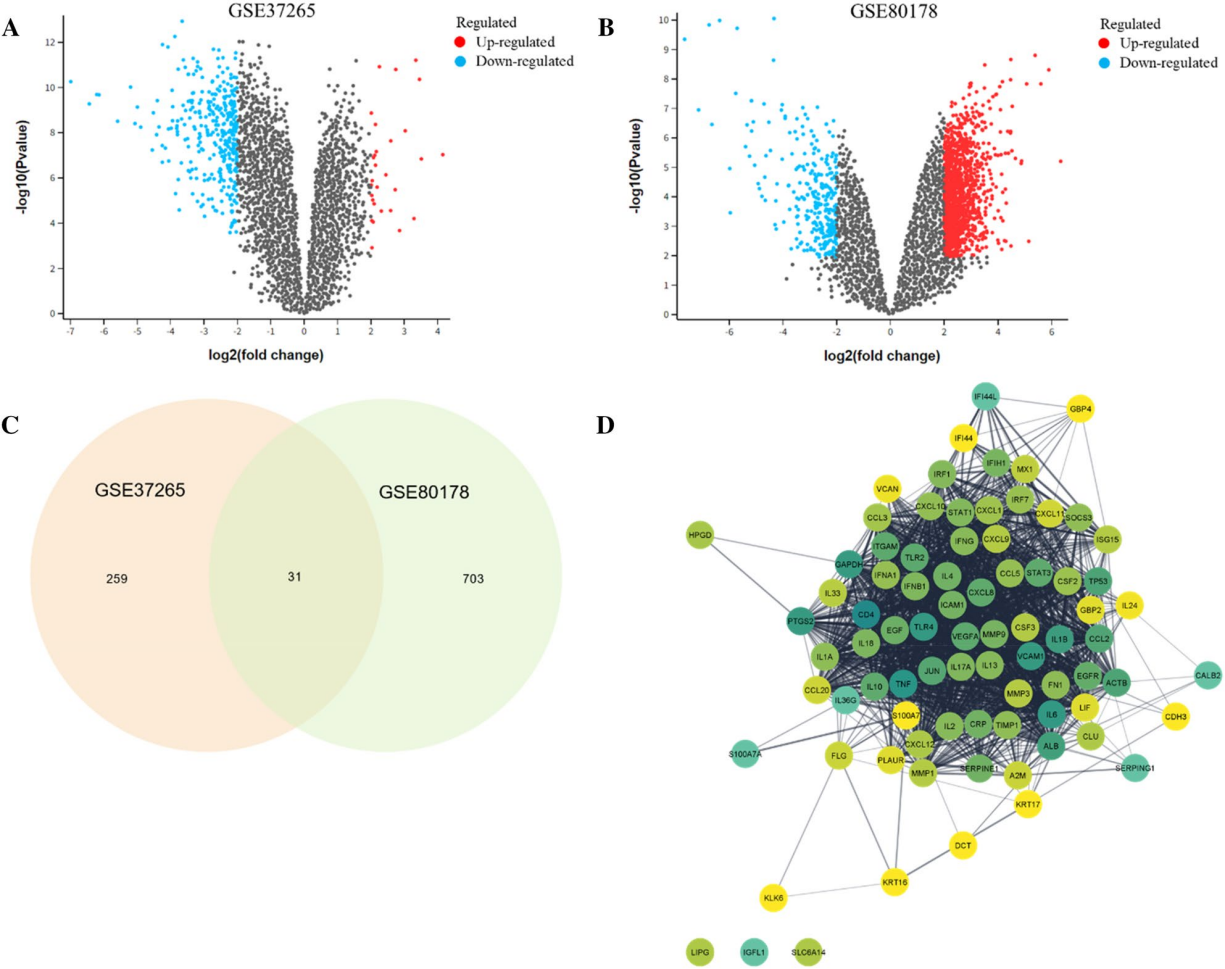
Detection of differentially expressed genes (DEGs) for diabetic foot ulcer. (**A**) A volcano plot corresponding to the GSE37265. (**B**) Volcano plot representing the identification of DEGs in the GSE80178 dataset. (**C**) Venn diagrams showing DEGs based on the intersection between GSE37265 and GSE80178 datasets. (**D**) Gene network expansion for the identified DEGs using STRING database. The color of the nodes reflects the degree of connectivity, with darker colors indicating a higher interaction score.

A total of 358 DEGs in the GSE37265 dataset were successfully identified, including 28 upregulated and 330 downregulated, as demonstrated in the volcano plots in Fig. 2A. Next, we identified the DEGs from the GSE80178 datasets between six DFUs and three normal skin tissues. This step, 734 DEGs were identified (175 upregulated genes and 559 downregulated genes), as illustrated in the volcano plots in Fig. 2B. To increase the stringency of the identified risk genes, all DEGs were collected based on the intersection of the analysis results of two datasets. Thirty-one overlapping genes were obtained from both groups, as shown in Fig. 2C and Supplementary Table S1.

### Biological DFU risk genes

**Figure 3:**
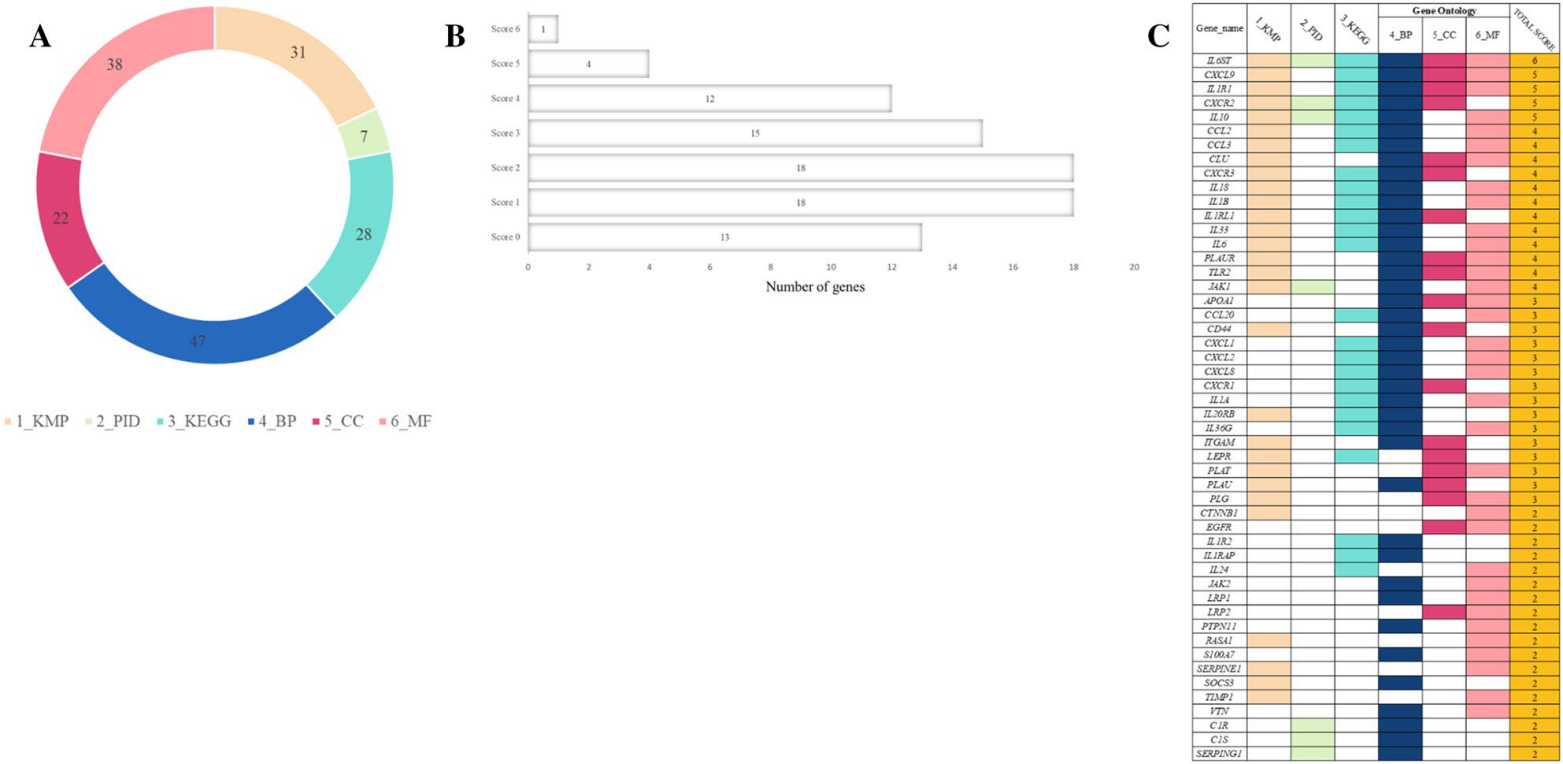
Prioritizing biological risk genes for diabetic foot ulcer (DFU) using a scoring system based on functional annotations. (**A**) A diagram pie chart shows the distribution score in each functional annotation for all 6 annotations. (**B**) An overview of gene score distribution for the 6 functional annotations. (**C**) Satisfied criteria for 50 biological DFU risk genes with a score of two or higher were indicated by the colors across each of the 6 functional annotations. A white box indicates the lack of functional annotations.

A strict functional annotation procedure was used to find biological DFU risk genes. To obtain more prospective drug target genes, we enlarge the networking of DEGs through the STRING database prior to identifying biological DFU risk genes. We set the threshold of 50 interactions, inputted 31 DEG from the phases before, and obtained 81 DEGs for further analysis (Fig. 2D; Supplementary Table S2). Furthermore, in order to prioritize genes for drug discovery for the present study, we utilized a scoring system previously employed in other research studies, as mentioned in the previous publications^16–19^. Following are the scoring results of the six functional annotations: (1) gene prioritized by Knockout mouse phenotype (KMP) (n = 31); (2) gene prioritized by Primary immunodeficiency (PID) 2019 (n = 7); (3) gene prioritized by Kyoto Encyclopedia of Genes and Genomes (KEGG) (n = 28); (4) gene prioritized by Biological processes (BP) (n = 47); (5) gene prioritized by Cellular components (CC) (n = 22); and (6) gene prioritized by Molecular functions (MF) (n = 38). Figure 3A and 3B shows a distribution score for each criterion. Ultimately, 50 biological DFU risk genes met the requirements with a score of 2 or higher. From further analysis of the gene scores, our findings revealed that the top five genes, *IL6ST, CXCL9, IL1R1, CXCR2,* and *IL10*, all having scores higher than 4 **(**Fig. 3C**)**.

### Discovery candidate drugs that potentially for DFU therapeutic

**Figure 4:**
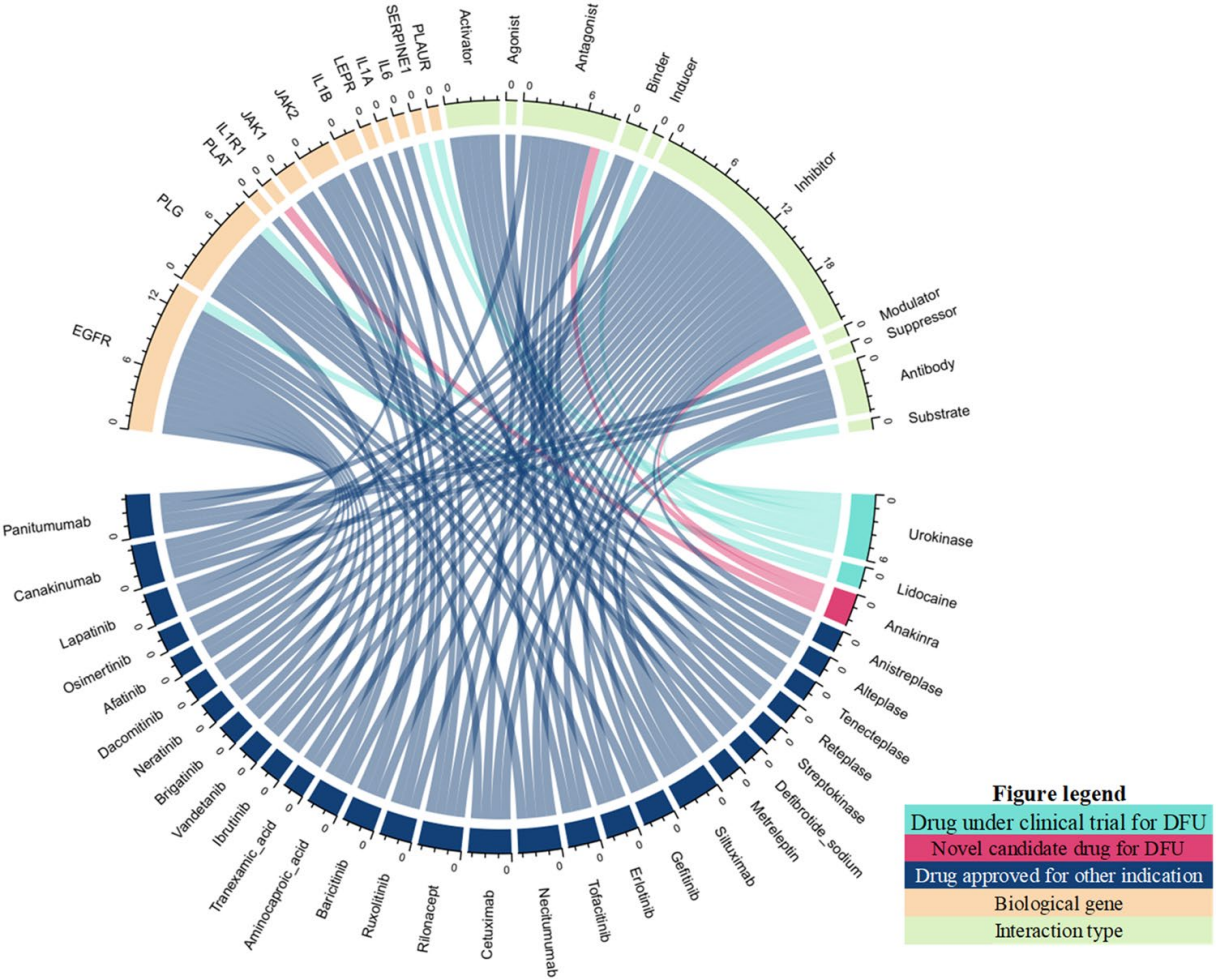
Chord diagram of the connections between prioritized genes against diabetic foot ulcer from our bioinformatic pipeline, and the indicated candidate drug(s) for each gene.

Next, we mapped 50 biological DFU risk genes into DGIdb (https://www.dgidb.org/search_interactions, accessed on November 12, 2022). We investigated whether gene profiles from biological DFU risk genes could be targeted by drugs with known interactions type. However, not all of the obtained biological DFU risk genes from the pipeline are druggable. Only 12 DFU-risk genes correspond to 31 drugs based on DGIdb (Supplementary Table S3). Hence, these drugs were considered as potential candidate drugs for DFU therapy. It is important to note among these 31 drugs, two drugs (urokinase; NCT01108120 and lidocaine; NCT04154046) were under clinical investigation for DFU, and 29 drugs were potential candidates drugs for DFU that have not been reported to DFU therapy **(**Fig. 4**)**. In particular, our study identified *IL1R1* overlapping anakinra was screened as a highly promising DFU target, since it also achieved a high systemic score in functional annotations. Our proof-of-principle study demonstrated that transcriptomic data not only can be utilized for biomarker discovery for DFUs, but it can also drive drug repurposing for this devastating disease.

## Discussion

Bioinformatics-driven drug repositioning is a method that uses computational techniques to identify new potential uses for existing drugs^20^. Drug repositioning is a promising strategy for treating DFU, a common complication of diabetes that can lead to amputation. By identifying existing drugs that could target the underlying molecular disease mechanisms, drug repositioning could provide an alternative, promising approach that quickly identifies and prioritize new potential treatment options for DFU patients. This study uses transcriptomic data to conduct a bioinformatics-based drug repositioning analysis for DFU. The study could analyze the gene expression profiles of tissue samples from patients with DFU and compare them to normal samples. This could help identify upregulated or downregulated genes in the ulcer tissue, which could suggest potential targets for drug repositioning. Our next step was prioritizing the DEGs for candidate drug prediction using a scoring system based on six functional annotations. Through this pipeline, we successfully identified 31 drugs targeting 12 genes, two of which (urokinase and lidocaine) are in clinical trials for DFU.

Our research identified the top 5 potential biomarkers in biological DFU risk genes: *IL6ST, CXCL9, IL1R1, CXCR2,* and *IL10*. *IL6ST,* also known as gp130, plays a key role in the inflammatory response and has been found to have higher expression in diabetic wounds^20–22^. *CXCL9* is a chemokine involved in the immune response that coordinates cells in the wound-healing process^23^. *CXCR2* is a chemokine receptor that is involved in inflammation. Keratinocytes and endothelial cells express *CXCR2* during wound healing in regions where epithelialization and neovascularization occur. Leukocyte recruitment, keratinocyte migration and proliferation, and angiogenesis are necessary for wound healing^24^. *IL10* is an anti-inflammatory cytokine that helps to regulate the immune response and reduce inflammation, with decreased levels in DFUs, suggesting that a lack of *IL10* may contribute to the progression of these wounds^25^. Lastly, *IL1R1* is a receptor for the pro-inflammatory cytokine *IL-1*. It is increased in DFUs, indicating an overactive *IL-1* response may contribute to the development of these wounds^26,27^. Of note, while our study focused specifically on gene expression differences between DFUs and healthy skin tissue, it is important to note that DFUs are a specific type of chronic wound with distinct characteristics and a unique pathophysiology compared to other types of wounds. Therefore, while the genes and pathways we identified may be involved in the wound healing process in general, we believe that our findings could find specific genes for DFUs and contribute to a better understanding of the molecular mechanisms underlying DFUs. Overall, the study suggests that five potential biomarkers in biological DFU risk genes: *IL6ST*, *CXCL9*, *IL1R1*, *CXCR2*, and *IL10* may serve as a useful biomarker for DFU and that targeting may be a potential therapeutic strategy for treating DFU.

Notably, among the top 5 potential biomarkers for DFU, we emphasize that only *IL1R1* is druggable and overlaps with the target of an existing drug, anakinra. Anakinra is a drug that is currently approved for the treatment of rheumatoid arthritis^28^. It is a recombinant human IL-1 receptor antagonist that works by binding to the IL-1 receptor and preventing the binding of pro- inflammatory IL-1 cytokines^29^. The *IL-1R1* signaling pathway is critical in the prevention of diabetic wound healing. Super-affinity IL-1Ra holds the potential for clinical translation in the treatment of chronic wounds and may also be used in other inflammatory conditions where *IL1R1* signaling plays a pathologic role^27^. This highlights the potential of *IL1R1* as a target for future drug development in treating DFU. However, it is important to consider the limitations of our bioinformatic approach, which requires clinical validation to verify the drugs and drug targets as a part of the drug discovery process that could ultimately lead to a cure for DFU. In addition, because a majority of the skin cells (> 95%) are keratinocytes, the bulk gene expression changes that we identify here as drug targets are contributed by this cell type. However, other cell types, such as immune cells, endothelial cells, and fibroblasts, play an important role in the wound-healing process. They could be potential druggable targets with further paired, cell-specific refined transcriptomic data^30,31^.

## Conclusion

In summary, a transcriptomic study has the potential to uncover new drug targets and repositioning opportunities for treating DFU. Specifically, five genes (*IL6ST, CXCL9, IL1R1, CXCR2,* and *IL10*) have been identified as high-priority targets for DFU drug repurposing. Among these targets, we focused on drugs targeting the gene *IL1R1* due to its high systemic score from our bioinformatic functional annotations. Additionally, *IL1R1* is the only gene among the five targets that overlaps with the target of an existing drug, Anakinra, further providing proof-of-concept for our approach. However, further clinical studies and validation are required to fully understand the mechanisms by which targeting *IL1R1* can be used to treat DFU as well as the validity of the identified drug regimens for DFU patients.

## Methods

### Identification of differentially expressed genes

The gene expression profiling datasets GSE37265 and GSE80178 were retrieved from the GEO database (https://www.ncbi.nlm.nih.gov/geo/, accessed on November 12, 2022). The GSE37265 expression profile comprised 14 DFU and 14 normal tissues, which were paired samples from the same patients, and was analyzed by the Affymetrix Human Genome U133 Plus 2.0 Array. The GSE80178 expression profile comprised six DFUs and three normal skin tissues, which were unpaired samples, and was analyzed by the Affymetrix Human Gene 2.0 ST Array. The downloaded data had already been normalized. Here, we use the limma package^32^ in R to identify DEGs. We applied a paired model matrix for the paired samples in GSE37265 to account for the correlation between the DFU and normal tissue samples from the same patients, and an unpaired model matrix for the unpaired samples in GSE80178 that did not account for pairing information. DEGs were screened using the following criteria: [log fold change (log FC)] > 2 and *p*-value 0.05. Only samples from human skin tissue were included in our study, and we ensured that the datasets had complete data for analysis and had obtained ethical approval. We also included DFU or normal samples and a control group without systemic or autoimmune diseases or a relevant family history, which met our necessary inclusion criteria. Finally, we visualized the DEGs that met the intersection between GSE37265 and GSE80178 using a Venn diagram.

### Expansion of the networking of differentially expressed genes by using the STRING Database

The DEG was enlarged using the STRING database to get more potential drug target genes. The STRING database (https://string-db.org, accessed on November 12, 2022) was created to integrate protein–protein interactions with functional connections related to protein expression^33^. We entered the list of DEGs chosen in the preceding phases and established the threshold of 50 interactions in order to increase the number of the initial set of DEGs. The rationale is a higher potential to discover new disease treatment targets with more disease-protein networks^34^.

### Prioritizing biological DFU risk genes

Next, we used the network-expanded list of DEGs, leveraging the STRING database for further functional annotation to explore deep insights into the pathogenesis of DFU and identify DFU therapeutic targets. The six functional annotations were applied to the filter of DEGs by a scoring system, with the criteria as follows: (1) KMP: To determine whether the gene contributes to certain mouse phenotypic diseases. Genes were prioritized using Mammalian Phenotype Ontology (MP) from WebGestalt (2019) with an FDR *q* < 0.05 and were considered significant^35^; (2) PID: DFU is associated with innate immune diseases. Noteworthy, genetic variants overlapping with PID genes may contribute to DFU pathogenesis. PID genes were collected from the 2019 Update of the IUIS Phenotypical Classification^36^. A hypergeometric test was used to conduct enrichment analysis on the data; the significance threshold for this step was a *p*-value < 0.05; 3) KEGG: to identify the molecular pathway^37^. KEGG prioritized genes in the Webgestalt^35^. The significance threshold was set at a *q*-value (FDR) < 0.05; 4) Gene Ontology (GO): Generally, GO categories can be broken down as follows: BP, CC, and MF to identify specific biological functions involved in DFU. A GO enrichment analysis was performed using from WebGestalt ^35^, and FDR *q* < 0.05 was used as a significance threshold. Genes scored more than equal to 2 were considered biological DFU-risk genes. Genes with higher annotation scores represent genes with a more prominent biological impact on DFU pathogenesis which we termed “biological risk genes”.

### Drug gene interactions for DFU

In order to identify DFU candidate drugs, we analyzed the set of biological DFU risk genes using the drug-gene interaction database (DGIdb) (https://www.dgidb.org/search_interactions). The DGIdb is an online database that compiles data on drug-gene interactions and druggable genes from articles, databases, and other online resources for drug discovery. The information about drugs, genes, and interactions is normalized and combined into conceptual groups^38^. From DGIdb, candidate drugs are selected based on FDA-approved drugs with clear interaction types.

### Statistical analysis

This study was conducted using R (version 4.2.1) for all statistical analyses. The *limma* package was used to identify DEGs^32^. The WebGestalt 2019 R package was used to perform over-representation analysis (ORA), including KMP, GO, and KEGG pathway enrichment analysis^35^. PID was analyzed using the hypergeometric test with a significance threshold of *p*-value < 0.05. The ggplot2 package (R v4.2.1)^39^ was used to visualize KMP, GO, and KEGG. The Venn diagram package (R v4.2.1)^40^ was applied to visualize all DEGs based on the intersection between GSE37265 and GSE80178. In addition, DFU candidate drugs were visualized using a chord diagram generated by the circlize package in R^41^.

## Supplementary Information

Supplementary Tables.

## Data Availability

All data generated or analyzed during this study are included in this article and its supplementary information files.

## Abbreviations

BP: Biological processes
CC: Cellular components
DEG: Differentially expressed genes
DFU: Diabetic foot ulcer
DGIdb: Drug-gene interaction database
DM: Diabetes melitus
GO: Gene Ontology
KEGG: Kyoto Encyclopedia of Genes and Genomes
KMP: Knockout mouse phenotype
Log FC: Log fold change
MF: Molecular functions
MP: Mammalian Phenotype Ontology
ORA: Over-representation analysis
PID: Primary immunodeficiency
