# Supplementary material for: Transcriptomics-driven drug repositioning for the treatment of diabetic foot ulcer
Source: Sci Rep. 2023 Jun 20;13:10032. doi: 10.1038/s41598-023-37120-1 (PMC10282022; doi:10.1038/s41598-023-37120-1)
Supplement: Supplementary file 1 — Supplementary Tables. [file 41598_2023_37120_MOESM1_ESM.docx]

**Drug Repositioning to Diabetic Foot Ulcer disease: a Transcriptomics Approach**

Wirawan Adikusuma^1,2,3†*,^ Zainul Amiruddin Zakaria^1*^, Lalu Muhammad Irham^4,5^, Baiq Leny Nopitasari^2^, Anna Pradiningsih^2^, Firdayani Firdayani^3^, Abdi Wira Septama^5^, Rockie Chong^6^

**Supplementary Materials**

Supplementary Table S1. Thirty-one DEGs were collected based on the intersection between GSE37265 and GSE80178.

Supplementary Table S2. DFU-related DEGs expanding by STRING database.

Supplementary Table S3. A list of candidate drugs for DFU based on DGIdb.

Supplementary Table S1. Thirty-one DEGs were collected based on the intersection between GSE37265 and GSE80178

| **GSE 80178** | | **GSE47265** | | **Intersection data** |
| --- | --- | --- | --- | --- |
| SPINK6 | UP | CRISP3 | UP | IGFL1 |
| S100A12 | UP | IGFL1 | UP | FLG |
| HEPHL1 | UP | LOC100653057 | UP | CALB2 |
| S100A9 | UP | SIAH3 | UP | HPGD |
| MMP1 | UP | FLG | UP | DCT |
| RHCG | UP | ST6GAL2 | UP | GBP4 |
| PI3 | UP | KRTAP3-2 | UP | VCAN |
| S100A8 | UP | ETNK2 | UP | IL24 |
| SPRR2B | UP | GYS2 | UP | GBP2 |
| DEFB103A | UP | WNK4 | UP | CLU |
| DEFB4A | UP | ARG1 | UP | SERPINE1 |
| IL1A | UP | ZNF365 | UP | CCL20 |
| PTHLH | UP | CALB2 | UP | SERPING1 |
| PRR9 | UP | HPGD | UP | PLAUR |
| SPRR2F | UP | WNT2B | UP | CDH3 |
| CLDN17 | UP | SAMD5 | UP | IL33 |
| KRT16 | UP | PRLR | UP | S100A7A |
| EPGN | UP | SLC6A4 | UP | IFI44 |
| LCE3A | UP | TOX3 | UP | CXCL8 |
| TMPRSS11D | UP | CTTNBP2 | UP | LIPG |
| KRT6A | UP | YOD1 | UP | KRT17 |
| UPP1 | UP | KRT31 | UP | IL1B |
| KRT6B | UP | ELOVL4 | UP | KRT16 |
| KRT17 | UP | TSPAN8 | UP | KLK6 |
| CXCL8 | UP | DAPL1 | UP | IFI44L |
| LIPG | UP | ADH7 | UP | IL36G |
| IGFL1 | UP | WNT4 | UP | SOCS3 |
| MIR31HG | UP | DCT | UP | SLC6A14 |
| S100A7A | UP | TNC | DOWN | S100A7 |
| CDA | UP | TLR7 | DOWN | CXCL9 |
| KRT75 | UP | ADAM12 | DOWN | MMP1 |
| FOSL1 | UP | ITGA4 | DOWN |  |
| SPRR1A | UP | IGSF6 | DOWN |  |
| AREG | UP | SLC7A7 | DOWN |  |
| KLK12 | UP | CTLA4 | DOWN |  |
| LCE3E | UP | FAM26F | DOWN |  |
| SPRR2C | UP | IL21R | DOWN |  |
| KRT16P3 | UP | ALOX12B | DOWN |  |
| S100A7 | UP | NR4A3 | DOWN |  |
| HK2 | UP | EMILIN2 | DOWN |  |
| C15orf48 | UP | CD1D | DOWN |  |
| SPRR1B | UP | ETV7 | DOWN |  |
| SCNN1D | UP | LOC106146153 | DOWN |  |
| CARHSP1 | UP | FBXO6 | DOWN |  |
| SPINK7 | UP | GPR68 | DOWN |  |
| PLAUR | UP | SLC51A | DOWN |  |
| IL36A | UP | SAA2 | DOWN |  |
| ODC1 | UP | MX1 | DOWN |  |
| ARG2 | UP | GBP4 | DOWN |  |
| KLK10 | UP | UBE2L6 | DOWN |  |
| ADM | UP | SEL1L2 | DOWN |  |
| IL24 | UP | CEMIP | DOWN |  |
| DDIT4 | UP | LOC100505984 | DOWN |  |
| DSC2 | UP | FPR3 | DOWN |  |
| AJAP1 | UP | AQP9 | DOWN |  |
| LINC00707 | UP | TNFRSF1B | DOWN |  |
| UPK1B | UP | VCAN | DOWN |  |
| SPRR4 | UP | P2RY6 | DOWN |  |
| GPRC5A | UP | RARRES3 | DOWN |  |
| IVL | UP | STOM | DOWN |  |
| BNIP3 | UP | ERP27 | DOWN |  |
| PPP4R4 | UP | C16orf54 | DOWN |  |
| CAMKK2 | UP | TARP | DOWN |  |
| IER3 | UP | PLA2G7 | DOWN |  |
| KRT16P2 | UP | HAVCR2 | DOWN |  |
| SLAMF7 | UP | IFITM1 | DOWN |  |
| HBEGF | UP | C1orf162 | DOWN |  |
| TREM1 | UP | CYP1B1 | DOWN |  |
| ACP7 | UP | IKZF1 | DOWN |  |
| LYPD5 | UP | CHI3L2 | DOWN |  |
| CHAC1 | UP | RGS1 | DOWN |  |
| SLPI | UP | TYROBP | DOWN |  |
| FCHSD1 | UP | PTPRC | DOWN |  |
| CALB2 | UP | COTL1 | DOWN |  |
| ANGPTL4 | UP | CD53 | DOWN |  |
| SCNN1G | UP | TAGAP | DOWN |  |
| IGHM | UP | PRKCB | DOWN |  |
| LCE3C | UP | COL4A1 | DOWN |  |
| TMPRSS4 | UP | C1R | DOWN |  |
| KLK9 | UP | LAP3 | DOWN |  |
| EIF3F | UP | KLRC2 | DOWN |  |
| PGLYRP4 | UP | ADAMDEC1 | DOWN |  |
| PHLDA2 | UP | ICAM1 | DOWN |  |
| RNF223 | UP | NAPSB | DOWN |  |
| KLK6 | UP | IRF1 | DOWN |  |
| QSOX1 | UP | IRF7 | DOWN |  |
| EIF4EBP1 | UP | FCGR2A | DOWN |  |
| SERPINE1 | UP | MPEG1 | DOWN |  |
| STK17A | UP | SLC2A14 | DOWN |  |
| ATP12A | UP | WISP1 | DOWN |  |
| FAM83A | UP | CTSS | DOWN |  |
| SERPINB1 | UP | C1QA | DOWN |  |
| TGM1 | UP | NDUFC2-KCTD14 | DOWN |  |
| KRT37 | UP | NCF2 | DOWN |  |
| CCL20 | UP | THEMIS2 | DOWN |  |
| PPIF | UP | SLC39A8 | DOWN |  |
| ANXA3 | UP | IL24 | DOWN |  |
| RNF185-AS1 | UP | GPR84 | DOWN |  |
| RHOF | UP | APOL3 | DOWN |  |
| IL1B | UP | IL11 | DOWN |  |
| ADAM23 | UP | HLA-DPA1 | DOWN |  |
| NPTX1 | UP | GBP2 | DOWN |  |
| NOCT | UP | GBP1 | DOWN |  |
| PAQR5 | UP | SNX20 | DOWN |  |
| CYTOR | UP | CR1 | DOWN |  |
| ANKRD31 | UP | LCP2 | DOWN |  |
| CDH3 | UP | CYTIP | DOWN |  |
| TNFRSF12A | UP | HLA-DMA | DOWN |  |
| CD24 | UP | CLU | DOWN |  |
| AKR1B10 | UP | XAF1 | DOWN |  |
| CLIC3 | UP | PTGES | DOWN |  |
| MXD1 | UP | SAA2-SAA4 | DOWN |  |
| ERO1A | UP | GPR65 | DOWN |  |
| KDM7A-DT | UP | SLC16A1 | DOWN |  |
| SERPINB3 | UP | LYN | DOWN |  |
| SOCS3 | UP | LOC101928195 | DOWN |  |
| ULBP1 | UP | C10orf99 | DOWN |  |
| ACP5 | UP | MARCH1 | DOWN |  |
| MGC4859 | UP | LOC100129518 | DOWN |  |
| GPX3 | UP | PIK3AP1 | DOWN |  |
| ANKRD37 | UP | BIRC3 | DOWN |  |
| TM4SF1 | UP | SECTM1 | DOWN |  |
| SESN2 | UP | LRG1 | DOWN |  |
| EEF1A2 | UP | LYZ | DOWN |  |
| ELF3 | UP | CLEC4A | DOWN |  |
| HILPDA | UP | IFIT2 | DOWN |  |
| CD68 | UP | STAT1 | DOWN |  |
| OR2L8 | UP | MICB | DOWN |  |
| SMOX | UP | GPR183 | DOWN |  |
| PFKFB3 | UP | PSMB9 | DOWN |  |
| RNF122 | UP | EPSTI1 | DOWN |  |
| DUSP5 | UP | TRNP1 | DOWN |  |
| SPRR2E | UP | KLHL6 | DOWN |  |
| FKBP1A-SDCBP2 | UP | LOC101928916 | DOWN |  |
| IL36G | UP | GNLY | DOWN |  |
| ADAP2 | UP | ISG20 | DOWN |  |
| TMEM54 | UP | SLAMF1 | DOWN |  |
| HCAR2 | UP | UCP2 | DOWN |  |
| TTC9 | UP | MNDA | DOWN |  |
| IL1RN | UP | C1QC | DOWN |  |
| ALOX12 | UP | FCER1G | DOWN |  |
| KCTD11 | UP | COL4A2 | DOWN |  |
| LCN2 | UP | HCLS1 | DOWN |  |
| PLA2G3 | UP | LYPD1 | DOWN |  |
| FUT2 | UP | IFITM2 | DOWN |  |
| PNLIPRP3 | UP | CYBB | DOWN |  |
| SULT2B1 | UP | SERPINE1 | DOWN |  |
| MALL | UP | SLC28A3 | DOWN |  |
| JUNB | UP | HSPA6 | DOWN |  |
| IGFN1 | UP | PLEK | DOWN |  |
| RHOD | UP | ZC3H12D | DOWN |  |
| TRPV3 | UP | APOL1 | DOWN |  |
| RIMS3 | UP | FAM65B | DOWN |  |
| TXN | UP | IRF8 | DOWN |  |
| GPR15LG | UP | OAS2 | DOWN |  |
| IL20 | UP | HCK | DOWN |  |
| RPS24 | UP | TAP1 | DOWN |  |
| SQSTM1 | UP | CD14 | DOWN |  |
| SPHK1 | UP | LILRA5 | DOWN |  |
| DEFB105B | UP | ST8SIA4 | DOWN |  |
| IGFL2 | UP | RARRES1 | DOWN |  |
| RNF39 | UP | MS4A7 | DOWN |  |
| TGFA | UP | C5AR1 | DOWN |  |
| FAM25A | UP | CD274 | DOWN |  |
| DSG3 | UP | THBS1 | DOWN |  |
| LDLR | UP | JAK3 | DOWN |  |
| ABCG4 | UP | SRGN | DOWN |  |
| SLC6A14 | UP | CD300LF | DOWN |  |
| ZNF426 | UP | DDX58 | DOWN |  |
| PRSS8 | UP | IFI35 | DOWN |  |
| CFL1 | UP | CCL20 | DOWN |  |
| NRBF2 | UP | SERPING1 | DOWN |  |
| ZBED2 | UP | PLAUR | DOWN |  |
| ETHE1 | UP | BATF2 | DOWN |  |
| IGFBP2 | UP | CEACAM7 | DOWN |  |
| CEP170 | DOWN | DDX60 | DOWN |  |
| MED1 | DOWN | GREM1 | DOWN |  |
| LINC00630 | DOWN | PXDN | DOWN |  |
| RPL30 | DOWN | FCN1 | DOWN |  |
| CNTLN | DOWN | TLR2 | DOWN |  |
| MS4A2 | DOWN | CCL4 | DOWN |  |
| SMG1P2 | DOWN | MS4A4A | DOWN |  |
| ZNF484 | DOWN | CD69 | DOWN |  |
| PROSER1 | DOWN | SLAMF8 | DOWN |  |
| POLK | DOWN | CCL18 | DOWN |  |
| PCID2 | DOWN | TNFSF13B | DOWN |  |
| MAP2K5 | DOWN | PDE4B | DOWN |  |
| TMEM237 | DOWN | CDH3 | DOWN |  |
| HSPBAP1 | DOWN | CD163 | DOWN |  |
| MYLIP | DOWN | TYMP | DOWN |  |
| SRGAP2C | DOWN | IL7R | DOWN |  |
| RECK | DOWN | FYB | DOWN |  |
| PIGK | DOWN | KLK7 | DOWN |  |
| TRAJ17 | DOWN | TIMP1 | DOWN |  |
| SNRPN | DOWN | SNX10 | DOWN |  |
| TAS2R31 | DOWN | IL33 | DOWN |  |
| RPS3A | DOWN | CD177 | DOWN |  |
| PARP14 | DOWN | FCGR1CP | DOWN |  |
| SLIT2 | DOWN | CCL19 | DOWN |  |
| KAT6B | DOWN | S100A7A | DOWN |  |
| TRPC1 | DOWN | ADGRE2 | DOWN |  |
| HLTF | DOWN | IFIT3 | DOWN |  |
| STS | DOWN | FPR1 | DOWN |  |
| APEX1 | DOWN | CSF2RB | DOWN |  |
| PTPN13 | DOWN | IFNG | DOWN |  |
| TUBE1 | DOWN | MUC4 | DOWN |  |
| FHL5 | DOWN | IFI30 | DOWN |  |
| MSH2 | DOWN | GPR171 | DOWN |  |
| TMEM150C | DOWN | C1QB | DOWN |  |
| NVL | DOWN | SPP1 | DOWN |  |
| FKSG29 | DOWN | SOCS1 | DOWN |  |
| SAMHD1 | DOWN | IFI44 | DOWN |  |
| FRA10AC1 | DOWN | XDH | DOWN |  |
| IDE | DOWN | MS4A1 | DOWN |  |
| ELMO1 | DOWN | CCR1 | DOWN |  |
| NBEAL1 | DOWN | LAMP3 | DOWN |  |
| STK32A | DOWN | KIAA0226L | DOWN |  |
| RBMS3 | DOWN | PLAT | DOWN |  |
| C5orf46 | DOWN | NRP2 | DOWN |  |
| SHLD2P3 | DOWN | ACOD1 | DOWN |  |
| CANX | DOWN | CCR7 | DOWN |  |
| KIAA0586 | DOWN | OASL | DOWN |  |
| NAF1 | DOWN | IFI27 | DOWN |  |
| MBTD1 | DOWN | EPPK1 | DOWN |  |
| TNRC6B | DOWN | SFRP4 | DOWN |  |
| GOLPH3L | DOWN | MIR155 | DOWN |  |
| SLC25A40 | DOWN | INHBA | DOWN |  |
| EMCN | DOWN | GBP5 | DOWN |  |
| PRKAR2B | DOWN | PTX3 | DOWN |  |
| SNORA16B | DOWN | CXCL13 | DOWN |  |
| AOPEP | DOWN | BST2 | DOWN |  |
| ITGBL1 | DOWN | CCL2 | DOWN |  |
| ZNF767P | DOWN | CXCL8 | DOWN |  |
| C11orf58 | DOWN | TFEC | DOWN |  |
| PHF14 | DOWN | SAMSN1 | DOWN |  |
| ZNF521 | DOWN | CHI3L1 | DOWN |  |
| ANTXR2 | DOWN | WARS | DOWN |  |
| HNRNPK | DOWN | SLC2A3 | DOWN |  |
| STARD9 | DOWN | LIPG | DOWN |  |
| THBS4 | DOWN | IL18BP | DOWN |  |
| KIAA0825 | DOWN | SERPINA1 | DOWN |  |
| GTF2IRD2B | DOWN | APOBEC3A_B | DOWN |  |
| CEP120 | DOWN | GLDC | DOWN |  |
| JAK2 | DOWN | KRT17 | DOWN |  |
| PAXBP1 | DOWN | IL1B | DOWN |  |
| ZNF718 | DOWN | TLR8 | DOWN |  |
| BBS5 | DOWN | TNFAIP6 | DOWN |  |
| IQGAP2 | DOWN | KRT16 | DOWN |  |
| SNHG1 | DOWN | CXCR4 | DOWN |  |
| PTPRM | DOWN | FCGR1B | DOWN |  |
| TLR3 | DOWN | IL12RB2 | DOWN |  |
| DENND4C | DOWN | PTGS2 | DOWN |  |
| COPZ1 | DOWN | FCGR3B | DOWN |  |
| SENP6 | DOWN | CCL3L3 | DOWN |  |
| OVCH2 | DOWN | CXCL3 | DOWN |  |
| TTC14 | DOWN | ISG15 | DOWN |  |
| PDS5B | DOWN | KLK6 | DOWN |  |
| SERPING1 | DOWN | IDO1 | DOWN |  |
| SEMA6D | DOWN | PI15 | DOWN |  |
| VPS13C | DOWN | IFI6 | DOWN |  |
| ANKRD44 | DOWN | IFI44L | DOWN |  |
| BDH2 | DOWN | IL36G | DOWN |  |
| ENTREP1 | DOWN | GZMB | DOWN |  |
| LINC00665 | DOWN | SELL | DOWN |  |
| RPL4 | DOWN | CMPK2 | DOWN |  |
| UGGT2 | DOWN | IL6 | DOWN |  |
| TCF4 | DOWN | LTF | DOWN |  |
| OR5P3 | DOWN | SELE | DOWN |  |
| ORMDL1 | DOWN | RSAD2 | DOWN |  |
| NPIPB9 | DOWN | UBD | DOWN |  |
| PDE5A | DOWN | CXCL2 | DOWN |  |
| TUT4 | DOWN | CXCL5 | DOWN |  |
| SEPSECS-AS1 | DOWN | CFB | DOWN |  |
| RPL7A | DOWN | SOCS3 | DOWN |  |
| LRCH1 | DOWN | BCL2A1 | DOWN |  |
| ZNF100 | DOWN | MMP12 | DOWN |  |
| CAPN3 | DOWN | LILRB2 | DOWN |  |
| TBCK | DOWN | CCL8 | DOWN |  |
| TNXB | DOWN | SLC6A14 | DOWN |  |
| COLEC12 | DOWN | MMP10 | DOWN |  |
| FAM221A | DOWN | S100A7 | DOWN |  |
| PARP4 | DOWN | CXCL6 | DOWN |  |
| KCTD12 | DOWN | KRT24 | DOWN |  |
| SPRED1 | DOWN | CXCL1 | DOWN |  |
| RALGAPA2 | DOWN | CXCL9 | DOWN |  |
| SLC10A5 | DOWN | CXCL10 | DOWN |  |
| ERVK13-1 | DOWN | DEFB4B | DOWN |  |
| GBP4 | DOWN | MMP3 | DOWN |  |
| PHC3 | DOWN | CXCL11 | DOWN |  |
| PIK3C2B | DOWN | MMP1 | DOWN |  |
| KMT2E | DOWN |  |  |  |
| ZEB1 | DOWN |  |  |  |
| DPH6 | DOWN |  |  |  |
| VEZF1 | DOWN |  |  |  |
| BCL11A | DOWN |  |  |  |
| FAM185A | DOWN |  |  |  |
| CLYBL | DOWN |  |  |  |
| MAGED2 | DOWN |  |  |  |
| CCDC144A | DOWN |  |  |  |
| NBEA | DOWN |  |  |  |
| ZNF790-AS1 | DOWN |  |  |  |
| NPY1R | DOWN |  |  |  |
| GULP1 | DOWN |  |  |  |
| ZNF704 | DOWN |  |  |  |
| NPIPB5 | DOWN |  |  |  |
| VN1R108P | DOWN |  |  |  |
| BHLHE41 | DOWN |  |  |  |
| MAMDC2 | DOWN |  |  |  |
| NHLRC2 | DOWN |  |  |  |
| NYNRIN | DOWN |  |  |  |
| OPHN1 | DOWN |  |  |  |
| TRPM7 | DOWN |  |  |  |
| OGN | DOWN |  |  |  |
| SNTB1 | DOWN |  |  |  |
| ZNHIT6 | DOWN |  |  |  |
| ZNF826P | DOWN |  |  |  |
| DYNC2H1 | DOWN |  |  |  |
| LRIG2 | DOWN |  |  |  |
| ANGEL2 | DOWN |  |  |  |
| CD44 | DOWN |  |  |  |
| CHD9 | DOWN |  |  |  |
| LINC01359 | DOWN |  |  |  |
| NEPRO | DOWN |  |  |  |
| EBF1 | DOWN |  |  |  |
| CILK1 | DOWN |  |  |  |
| OR2A4 | DOWN |  |  |  |
| CACNA2D1 | DOWN |  |  |  |
| MFSD4A-AS1 | DOWN |  |  |  |
| BLMH | DOWN |  |  |  |
| UBN2 | DOWN |  |  |  |
| TIA1 | DOWN |  |  |  |
| PNISR | DOWN |  |  |  |
| EFCAB7 | DOWN |  |  |  |
| MICU3 | DOWN |  |  |  |
| CTPS2 | DOWN |  |  |  |
| CARF | DOWN |  |  |  |
| CDKAL1 | DOWN |  |  |  |
| MTR | DOWN |  |  |  |
| GXYLT2 | DOWN |  |  |  |
| PM20D2 | DOWN |  |  |  |
| TAS2R4 | DOWN |  |  |  |
| TRANK1 | DOWN |  |  |  |
| PARK7 | DOWN |  |  |  |
| PDGFRB | DOWN |  |  |  |
| FAXDC2 | DOWN |  |  |  |
| ARL17B | DOWN |  |  |  |
| LOC100506124 | DOWN |  |  |  |
| LINC01921 | DOWN |  |  |  |
| PTGER3 | DOWN |  |  |  |
| MSH5-SAPCD1 | DOWN |  |  |  |
| SRSF1 | DOWN |  |  |  |
| GEN1 | DOWN |  |  |  |
| HNRNPD | DOWN |  |  |  |
| CYP3A5 | DOWN |  |  |  |
| DBT | DOWN |  |  |  |
| LRP6 | DOWN |  |  |  |
| HIBCH | DOWN |  |  |  |
| CENPC | DOWN |  |  |  |
| PKD1P1 | DOWN |  |  |  |
| SLC16A14 | DOWN |  |  |  |
| SNORA74A | DOWN |  |  |  |
| HCP5 | DOWN |  |  |  |
| ANGPTL7 | DOWN |  |  |  |
| LINC01876 | DOWN |  |  |  |
| PARM1 | DOWN |  |  |  |
| DTWD1 | DOWN |  |  |  |
| KAT2A | DOWN |  |  |  |
| MAP3K1 | DOWN |  |  |  |
| CEP162 | DOWN |  |  |  |
| NONO | DOWN |  |  |  |
| EOLA2-DT | DOWN |  |  |  |
| ZNF37BP | DOWN |  |  |  |
| PAMR1 | DOWN |  |  |  |
| NFIA | DOWN |  |  |  |
| ATG2B | DOWN |  |  |  |
| FMOD | DOWN |  |  |  |
| AKT3 | DOWN |  |  |  |
| PDIA3P1 | DOWN |  |  |  |
| NSL1 | DOWN |  |  |  |
| GABPB1-AS1 | DOWN |  |  |  |
| MTERF2 | DOWN |  |  |  |
| HPGD | DOWN |  |  |  |
| ETV6 | DOWN |  |  |  |
| SDHAP4 | DOWN |  |  |  |
| TFDP2 | DOWN |  |  |  |
| PWAR5 | DOWN |  |  |  |
| STARD7 | DOWN |  |  |  |
| PPIP5K2 | DOWN |  |  |  |
| NHLRC3 | DOWN |  |  |  |
| PRKN | DOWN |  |  |  |
| GNPDA2 | DOWN |  |  |  |
| GSTM5 | DOWN |  |  |  |
| P2RY14 | DOWN |  |  |  |
| KRIT1 | DOWN |  |  |  |
| ARID4A | DOWN |  |  |  |
| LUC7L3 | DOWN |  |  |  |
| PA2G4P4 | DOWN |  |  |  |
| EPB41L2 | DOWN |  |  |  |
| CHD6 | DOWN |  |  |  |
| TOGARAM1 | DOWN |  |  |  |
| JPX | DOWN |  |  |  |
| FGF7 | DOWN |  |  |  |
| RAVER2 | DOWN |  |  |  |
| KMT2A | DOWN |  |  |  |
| SLC25A3 | DOWN |  |  |  |
| HERC2P9 | DOWN |  |  |  |
| RSL1D1 | DOWN |  |  |  |
| SAMD9L | DOWN |  |  |  |
| PREX2 | DOWN |  |  |  |
| ATM | DOWN |  |  |  |
| LGR5 | DOWN |  |  |  |
| MAGOHB | DOWN |  |  |  |
| CHPT1 | DOWN |  |  |  |
| PTGIS | DOWN |  |  |  |
| RRP7BP | DOWN |  |  |  |
| REV3L | DOWN |  |  |  |
| AHSA2P | DOWN |  |  |  |
| CEP126 | DOWN |  |  |  |
| PARP8 | DOWN |  |  |  |
| PCDHB14 | DOWN |  |  |  |
| UTRN | DOWN |  |  |  |
| ZNF507 | DOWN |  |  |  |
| SRGAP2 | DOWN |  |  |  |
| CRTAP | DOWN |  |  |  |
| GNB4 | DOWN |  |  |  |
| PAN2 | DOWN |  |  |  |
| LINC02984 | DOWN |  |  |  |
| L3MBTL4 | DOWN |  |  |  |
| HPGDS | DOWN |  |  |  |
| LIAS | DOWN |  |  |  |
| RCOR3 | DOWN |  |  |  |
| LRRC39 | DOWN |  |  |  |
| KAT7 | DOWN |  |  |  |
| WIF1 | DOWN |  |  |  |
| TRAK2 | DOWN |  |  |  |
| ZNF658B | DOWN |  |  |  |
| XNDC1N | DOWN |  |  |  |
| NUAK1 | DOWN |  |  |  |
| SMC5 | DOWN |  |  |  |
| PRH1-PRR4 | DOWN |  |  |  |
| WRN | DOWN |  |  |  |
| LOC100190986 | DOWN |  |  |  |
| ANKRD49 | DOWN |  |  |  |
| SULT1E1 | DOWN |  |  |  |
| ARHGAP42 | DOWN |  |  |  |
| HMCN1 | DOWN |  |  |  |
| LHFPL6 | DOWN |  |  |  |
| HINT1 | DOWN |  |  |  |
| KCNJ8 | DOWN |  |  |  |
| DIAPH2 | DOWN |  |  |  |
| RAD50 | DOWN |  |  |  |
| MIS18BP1 | DOWN |  |  |  |
| PMS2P1 | DOWN |  |  |  |
| NEK3 | DOWN |  |  |  |
| HERC2P2 | DOWN |  |  |  |
| SMARCAD1 | DOWN |  |  |  |
| BGN | DOWN |  |  |  |
| FAM66C | DOWN |  |  |  |
| ZZZ3 | DOWN |  |  |  |
| ZNF280D | DOWN |  |  |  |
| IFT74 | DOWN |  |  |  |
| ANKRD36BP2 | DOWN |  |  |  |
| CEP290 | DOWN |  |  |  |
| GATM | DOWN |  |  |  |
| ASPA | DOWN |  |  |  |
| ANKRD20A2P | DOWN |  |  |  |
| PTAR1 | DOWN |  |  |  |
| ZNF280C | DOWN |  |  |  |
| ARMCX4 | DOWN |  |  |  |
| ZNF480 | DOWN |  |  |  |
| MMP2 | DOWN |  |  |  |
| HERC2P4 | DOWN |  |  |  |
| TFAP2B | DOWN |  |  |  |
| TAS2R14 | DOWN |  |  |  |
| ZMAT1 | DOWN |  |  |  |
| MCM8 | DOWN |  |  |  |
| ABCC5 | DOWN |  |  |  |
| GBP2 | DOWN |  |  |  |
| CES4A | DOWN |  |  |  |
| AADAC | DOWN |  |  |  |
| PWARSN | DOWN |  |  |  |
| TBC1D32 | DOWN |  |  |  |
| PLCG2 | DOWN |  |  |  |
| LOC105369477 | DOWN |  |  |  |
| ZNF383 | DOWN |  |  |  |
| FBLN5 | DOWN |  |  |  |
| ZKSCAN8 | DOWN |  |  |  |
| PDGFD | DOWN |  |  |  |
| ANK2 | DOWN |  |  |  |
| IFI44L | DOWN |  |  |  |
| MAP1B | DOWN |  |  |  |
| MACROD1 | DOWN |  |  |  |
| TAS2R3 | DOWN |  |  |  |
| SRSF11 | DOWN |  |  |  |
| MCOLN3 | DOWN |  |  |  |
| MAP4K3 | DOWN |  |  |  |
| ZNF25 | DOWN |  |  |  |
| PIBF1 | DOWN |  |  |  |
| TMEM9B-AS1 | DOWN |  |  |  |
| PSIP1 | DOWN |  |  |  |
| DLEU2L | DOWN |  |  |  |
| DEGS1 | DOWN |  |  |  |
| ZBTB20 | DOWN |  |  |  |
| TARBP1 | DOWN |  |  |  |
| NAPEPLD | DOWN |  |  |  |
| FAM117B | DOWN |  |  |  |
| LOC389765 | DOWN |  |  |  |
| LONRF1 | DOWN |  |  |  |
| TANC1 | DOWN |  |  |  |
| PCDHB13 | DOWN |  |  |  |
| LINC01355 | DOWN |  |  |  |
| ZDHHC11 | DOWN |  |  |  |
| FRG1HP | DOWN |  |  |  |
| UPK3B | DOWN |  |  |  |
| SPATA6 | DOWN |  |  |  |
| BTC | DOWN |  |  |  |
| PROS1 | DOWN |  |  |  |
| PLEKHH2 | DOWN |  |  |  |
| GUSBP16 | DOWN |  |  |  |
| RNF125 | DOWN |  |  |  |
| FCER1A | DOWN |  |  |  |
| PCDH18 | DOWN |  |  |  |
| C3 | DOWN |  |  |  |
| BDNF-AS | DOWN |  |  |  |
| CRYBB2P1 | DOWN |  |  |  |
| LRRCC1 | DOWN |  |  |  |
| RNPC3 | DOWN |  |  |  |
| RPGRIP1L | DOWN |  |  |  |
| GATA3 | DOWN |  |  |  |
| FABP7 | DOWN |  |  |  |
| SEPTIN2 | DOWN |  |  |  |
| SEPTIN7P2 | DOWN |  |  |  |
| KDM2B | DOWN |  |  |  |
| SEMA3D | DOWN |  |  |  |
| FNTA | DOWN |  |  |  |
| ZBTB41 | DOWN |  |  |  |
| FTX | DOWN |  |  |  |
| ATP13A4 | DOWN |  |  |  |
| TAS2R20 | DOWN |  |  |  |
| IGFL4 | DOWN |  |  |  |
| DPP4 | DOWN |  |  |  |
| IL7 | DOWN |  |  |  |
| LRP4 | DOWN |  |  |  |
| DDX5 | DOWN |  |  |  |
| PRELP | DOWN |  |  |  |
| PSMD6-AS2 | DOWN |  |  |  |
| FNDC1 | DOWN |  |  |  |
| TSPAN7 | DOWN |  |  |  |
| GUSBP2 | DOWN |  |  |  |
| CLU | DOWN |  |  |  |
| GCSHP3 | DOWN |  |  |  |
| FGL2 | DOWN |  |  |  |
| NFE2L3 | DOWN |  |  |  |
| TNFRSF19 | DOWN |  |  |  |
| LOC283299 | DOWN |  |  |  |
| NKTR | DOWN |  |  |  |
| ANKRD20A5P | DOWN |  |  |  |
| ZNF302 | DOWN |  |  |  |
| NAIP | DOWN |  |  |  |
| TARDBP | DOWN |  |  |  |
| GABRP | DOWN |  |  |  |
| NRN1 | DOWN |  |  |  |
| CP | DOWN |  |  |  |
| GUSBP1 | DOWN |  |  |  |
| ENPP2 | DOWN |  |  |  |
| FGFR2 | DOWN |  |  |  |
| KLHDC1 | DOWN |  |  |  |
| KRT73 | DOWN |  |  |  |
| GAN | DOWN |  |  |  |
| CYP2J2 | DOWN |  |  |  |
| SCARA3 | DOWN |  |  |  |
| LOC100506606 | DOWN |  |  |  |
| ZNF141 | DOWN |  |  |  |
| LIPM | DOWN |  |  |  |
| CTSO | DOWN |  |  |  |
| PDK4 | DOWN |  |  |  |
| P2RX7 | DOWN |  |  |  |
| CDC14A | DOWN |  |  |  |
| ARHGAP29 | DOWN |  |  |  |
| RIC3 | DOWN |  |  |  |
| ZC3H6 | DOWN |  |  |  |
| EPS8 | DOWN |  |  |  |
| CNTNAP3 | DOWN |  |  |  |
| SNORD114-3 | DOWN |  |  |  |
| ARL6IP5 | DOWN |  |  |  |
| JAM2 | DOWN |  |  |  |
| BBS2 | DOWN |  |  |  |
| MEOX2 | DOWN |  |  |  |
| LINC01422 | DOWN |  |  |  |
| PSMB2 | DOWN |  |  |  |
| ACP3 | DOWN |  |  |  |
| PMS2P9 | DOWN |  |  |  |
| MIR99AHG | DOWN |  |  |  |
| PRG2 | DOWN |  |  |  |
| IL33 | DOWN |  |  |  |
| CNTNAP3P2 | DOWN |  |  |  |
| ARHGEF26 | DOWN |  |  |  |
| ARHGEF6 | DOWN |  |  |  |
| SLC12A2 | DOWN |  |  |  |
| CMAHP | DOWN |  |  |  |
| PGAP1 | DOWN |  |  |  |
| DAB2 | DOWN |  |  |  |
| TMEM47 | DOWN |  |  |  |
| MYCBP2 | DOWN |  |  |  |
| VCAN | DOWN |  |  |  |
| SERTAD4 | DOWN |  |  |  |
| DPT | DOWN |  |  |  |
| GABRE | DOWN |  |  |  |
| MYH11 | DOWN |  |  |  |
| OVOS2 | DOWN |  |  |  |
| BOC | DOWN |  |  |  |
| TIMM23B | DOWN |  |  |  |
| GLYATL2 | DOWN |  |  |  |
| F13A1 | DOWN |  |  |  |
| LOC100289230 | DOWN |  |  |  |
| IFI44 | DOWN |  |  |  |
| SVEP1 | DOWN |  |  |  |
| COL14A1 | DOWN |  |  |  |
| ANKRD10-IT1 | DOWN |  |  |  |
| PHF3 | DOWN |  |  |  |
| ADD3 | DOWN |  |  |  |
| RPS27A | DOWN |  |  |  |
| LYVE1 | DOWN |  |  |  |
| CA6 | DOWN |  |  |  |
| ANTXR1 | DOWN |  |  |  |
| DCLK1 | DOWN |  |  |  |
| LINC03007 | DOWN |  |  |  |
| PLCB4 | DOWN |  |  |  |
| ALDH1A1 | DOWN |  |  |  |
| HNRNPU | DOWN |  |  |  |
| LAMA2 | DOWN |  |  |  |
| ANXA9 | DOWN |  |  |  |
| LRIG3 | DOWN |  |  |  |
| ARHGEF34P | DOWN |  |  |  |
| LOC107914879 | DOWN |  |  |  |
| TAS2R19 | DOWN |  |  |  |
| SLIT3 | DOWN |  |  |  |
| MIR570HG | DOWN |  |  |  |
| CDHR1 | DOWN |  |  |  |
| FBN1 | DOWN |  |  |  |
| ZC2HC1A | DOWN |  |  |  |
| ZNF253 | DOWN |  |  |  |
| CD34 | DOWN |  |  |  |
| CDON | DOWN |  |  |  |
| MMP7 | DOWN |  |  |  |
| SPARCL1 | DOWN |  |  |  |
| ANAPC5 | DOWN |  |  |  |
| CTSK | DOWN |  |  |  |
| SRGAP2B | DOWN |  |  |  |
| FRZB | DOWN |  |  |  |
| TNKS | DOWN |  |  |  |
| RPL6 | DOWN |  |  |  |
| SEPTIN7P14 | DOWN |  |  |  |
| PLSCR4 | DOWN |  |  |  |
| TTC21B | DOWN |  |  |  |
| KIT | DOWN |  |  |  |
| NEO1 | DOWN |  |  |  |
| SCGB1B2P | DOWN |  |  |  |
| DCN | DOWN |  |  |  |
| ABCA10 | DOWN |  |  |  |
| APRG1 | DOWN |  |  |  |
| CHL1 | DOWN |  |  |  |
| CCDC146 | DOWN |  |  |  |
| CD302 | DOWN |  |  |  |
| MRTFB | DOWN |  |  |  |
| SH3BP5-AS1 | DOWN |  |  |  |
| SLC25A27 | DOWN |  |  |  |
| NPIPB3 | DOWN |  |  |  |
| OMD | DOWN |  |  |  |
| C7 | DOWN |  |  |  |
| TXNIP | DOWN |  |  |  |
| CACHD1 | DOWN |  |  |  |
| SEPTIN7P13 | DOWN |  |  |  |
| ABCA9 | DOWN |  |  |  |
| ABCC9 | DOWN |  |  |  |
| MFAP4 | DOWN |  |  |  |
| TMEM116 | DOWN |  |  |  |
| PIK3R1 | DOWN |  |  |  |
| PLLP | DOWN |  |  |  |
| CCDC80 | DOWN |  |  |  |
| RPL23AP32 | DOWN |  |  |  |
| ECM2 | DOWN |  |  |  |
| ACADSB | DOWN |  |  |  |
| FABP4 | DOWN |  |  |  |
| ANKRD36B | DOWN |  |  |  |
| TGFBR3 | DOWN |  |  |  |
| FLG | DOWN |  |  |  |
| NFIB | DOWN |  |  |  |
| CYBRD1 | DOWN |  |  |  |
| GPR34 | DOWN |  |  |  |
| EEF2K | DOWN |  |  |  |
| KANSL1L-AS1 | DOWN |  |  |  |
| MXRA5 | DOWN |  |  |  |
| SERPINF1 | DOWN |  |  |  |
| CLDN10 | DOWN |  |  |  |
| ZNF652 | DOWN |  |  |  |
| AADACL2 | DOWN |  |  |  |
| NFIX | DOWN |  |  |  |
| SYNPO2 | DOWN |  |  |  |
| SFRP2 | DOWN |  |  |  |
| LIFR | DOWN |  |  |  |
| ABHD12B | DOWN |  |  |  |
| CXCL9 | DOWN |  |  |  |
| BMS1P1 | DOWN |  |  |  |
| TEX9 | DOWN |  |  |  |
| CHRDL1 | DOWN |  |  |  |
| N4BP2L2-IT2 | DOWN |  |  |  |
| OR5P2 | DOWN |  |  |  |
| MGP | DOWN |  |  |  |
| LAMB4 | DOWN |  |  |  |
| CFH | DOWN |  |  |  |
| CLEC2B | DOWN |  |  |  |
| GUSBP3 | DOWN |  |  |  |
| ABCA8 | DOWN |  |  |  |
| SERPINA12 | DOWN |  |  |  |
| SCGB1D2 | DOWN |  |  |  |
| PRG4 | DOWN |  |  |  |
| ABCA6 | DOWN |  |  |  |
| TYRP1 | DOWN |  |  |  |
| LINC00342 | DOWN |  |  |  |
| IL37 | DOWN |  |  |  |
| PIK3C2G | DOWN |  |  |  |
| DCT | DOWN |  |  |  |
| APOD | DOWN |  |  |  |
| ANGPTL1 | DOWN |  |  |  |
| XIST | DOWN |  |  |  |
| ABI3BP | DOWN |  |  |  |
| CLEC2A | DOWN |  |  |  |
| VPS51 | DOWN |  |  |  |
| PIP | DOWN |  |  |  |
| FLG2 | DOWN |  |  |  |
| DCD | DOWN |  |  |  |
| SCGB2A2 | DOWN |  |  |  |
| KRT2 | DOWN |  |  |  |

Supplementary Table S2. DFU-related DEGs expanding by STRING database.

| #node | identifier |
| --- | --- |
| ACKR1 | 9606.ENSP00000357103 |
| APOA1 | 9606.ENSP00000236850 |
| C1R | 9606.ENSP00000438615 |
| C1S | 9606.ENSP00000385035 |
| CALB2 | 9606.ENSP00000307508 |
| CASP1 | 9606.ENSP00000433138 |
| CCL2 | 9606.ENSP00000225831 |
| CCL20 | 9606.ENSP00000351671 |
| CCL3 | 9606.ENSP00000477908 |
| CCL4 | 9606.ENSP00000482259 |
| CD44 | 9606.ENSP00000398632 |
| CDH3 | 9606.ENSP00000264012 |
| CLU | 9606.ENSP00000315130 |
| CTNNB1 | 9606.ENSP00000344456 |
| CUL5 | 9606.ENSP00000376808 |
| CXCL1 | 9606.ENSP00000379110 |
| CXCL2 | 9606.ENSP00000427279 |
| CXCL8 | 9606.ENSP00000306512 |
| CXCL9 | 9606.ENSP00000354901 |
| CXCR1 | 9606.ENSP00000295683 |
| CXCR2 | 9606.ENSP00000319635 |
| CXCR3 | 9606.ENSP00000362795 |
| DCT | 9606.ENSP00000392762 |
| EGFR | 9606.ENSP00000275493 |
| ENSP00000383715 | 9606.ENSP00000383715 |
| FLG | 9606.ENSP00000357789 |
| GBP2 | 9606.ENSP00000359497 |
| GBP4 | 9606.ENSP00000359490 |
| GPR29 | 9606.ENSP00000343952 |
| HPGD | 9606.ENSP00000296522 |
| IFI44 | 9606.ENSP00000359783 |
| IFI44L | 9606.ENSP00000359787 |
| IGFL1 | 9606.ENSP00000415823 |
| IL10 | 9606.ENSP00000412237 |
| IL18 | 9606.ENSP00000280357 |
| IL1A | 9606.ENSP00000263339 |
| IL1B | 9606.ENSP00000263341 |
| IL1R1 | 9606.ENSP00000386380 |
| IL1R2 | 9606.ENSP00000330959 |
| IL1RAP | 9606.ENSP00000314807 |
| IL1RL1 | 9606.ENSP00000233954 |
| IL20RA | 9606.ENSP00000314976 |
| IL20RB | 9606.ENSP00000328133 |
| IL22RA1 | 9606.ENSP00000270800 |
| IL24 | 9606.ENSP00000375795 |
| IL33 | 9606.ENSP00000370842 |
| IL36G | 9606.ENSP00000259205 |
| IL6 | 9606.ENSP00000385675 |
| IL6ST | 9606.ENSP00000370698 |
| IRS1 | 9606.ENSP00000304895 |
| ITGAM | 9606.ENSP00000441691 |
| JAK1 | 9606.ENSP00000343204 |
| JAK2 | 9606.ENSP00000371067 |
| KLK6 | 9606.ENSP00000366047 |
| KRT16 | 9606.ENSP00000301653 |
| KRT17 | 9606.ENSP00000308452 |
| LEPR | 9606.ENSP00000330393 |
| LIPG | 9606.ENSP00000261292 |
| LRP1 | 9606.ENSP00000243077 |
| LRP2 | 9606.ENSP00000263816 |
| MMP1 | 9606.ENSP00000322788 |
| PLAT | 9606.ENSP00000220809 |
| PLAU | 9606.ENSP00000361850 |
| PLAUR | 9606.ENSP00000339328 |
| PLG | 9606.ENSP00000308938 |
| PTPN11 | 9606.ENSP00000340944 |
| RASA1 | 9606.ENSP00000274376 |
| S100A7 | 9606.ENSP00000357712 |
| S100A7A | 9606.ENSP00000357718 |
| SDC3 | 9606.ENSP00000344468 |
| SERPINE1 | 9606.ENSP00000223095 |
| SERPING1 | 9606.ENSP00000278407 |
| SLC6A14 | 9606.ENSP00000470801 |
| SOCS3 | 9606.ENSP00000330341 |
| TCEB1 | 9606.ENSP00000478121 |
| TCEB2 | 9606.ENSP00000262306 |
| TIMP1 | 9606.ENSP00000218388 |
| TLR2 | 9606.ENSP00000260010 |
| VCAN | 9606.ENSP00000265077 |
| VTN | 9606.ENSP00000226218 |
| XRCC6 | 9606.ENSP00000352257 |

Supplementary Table S3. A list of candidate drugs for DFU based on DGIdb.

| **gene** | **drug** | **interaction_types** | **Status** | **Phase** | **NCT Number/ PMID** |
| --- | --- | --- | --- | --- | --- |
| PLG | UROKINASE | activator | Unknown | Phase 2 | NCT01108120 |
| PLG | ANISTREPLASE | activator |  |  |  |
| PLG | ALTEPLASE | activator |  |  |  |
| PLG | TENECTEPLASE | activator |  |  |  |
| PLG | RETEPLASE | activator |  |  |  |
| PLG | STREPTOKINASE | activator |  |  |  |
| PLG | DEFIBROTIDE SODIUM | activator |  |  |  |
| LEPR | METRELEPTIN | agonist |  |  |  |
| EGFR | LIDOCAINE | antagonist | Completed | Not Applicable | NCT04154046 |
| IL6 | SILTUXIMAB | antagonist\|antibody\|inhibitor |  |  |  |
| EGFR | GEFITINIB | antagonist\|inhibitor |  |  |  |
| EGFR | ERLOTINIB | antagonist\|inhibitor |  |  |  |
| JAK2 | TOFACITINIB | antagonist\|inhibitor |  |  |  |
| EGFR | NECITUMUMAB | antagonist\|inhibitor\|antibody |  |  |  |
| EGFR | CETUXIMAB | antagonist\|inhibitor\|antibody |  |  |  |
| IL1A | RILONACEPT | binder |  |  |  |
| IL1B | RILONACEPT | binder\|inhibitor |  |  |  |
| SERPINE1 | UROKINASE | inducer\|substrate |  |  |  |
| JAK1 | TOFACITINIB CITRATE | inhibitor |  |  |  |
| JAK1 | RUXOLITINIB | inhibitor |  |  |  |
| JAK1 | BARICITINIB | inhibitor |  |  |  |
| JAK1 | RUXOLITINIB PHOSPHATE | inhibitor |  |  |  |
| PLAT | AMINOCAPROIC ACID | inhibitor |  |  |  |
| PLG | TRANEXAMIC ACID | inhibitor |  |  |  |
| PLG | AMINOCAPROIC ACID | inhibitor |  |  |  |
| EGFR | IBRUTINIB | inhibitor |  |  |  |
| EGFR | VANDETANIB | inhibitor |  |  |  |
| EGFR | BRIGATINIB | inhibitor |  |  |  |
| EGFR | ERLOTINIB HYDROCHLORIDE | inhibitor |  |  |  |
| EGFR | NERATINIB | inhibitor |  |  |  |
| EGFR | DACOMITINIB | inhibitor |  |  |  |
| EGFR | AFATINIB | inhibitor |  |  |  |
| EGFR | OSIMERTINIB | inhibitor |  |  |  |
| EGFR | AFATINIB DIMALEATE | inhibitor |  |  |  |
| EGFR | OSIMERTINIB MESYLATE | inhibitor |  |  |  |
| EGFR | LAPATINIB DITOSYLATE | inhibitor |  |  |  |
| JAK2 | TOFACITINIB CITRATE | inhibitor |  |  |  |
| JAK2 | RUXOLITINIB | inhibitor |  |  |  |
| JAK2 | BARICITINIB | inhibitor |  |  |  |
| JAK2 | RUXOLITINIB PHOSPHATE | inhibitor |  |  |  |
| IL1R1 | ANAKINRA | inhibitor\|antagonist |  |  |  |
| EGFR | LAPATINIB | inhibitor\|antagonist |  |  |  |
| IL1B | CANAKINUMAB | inhibitor\|binder\|antibody |  |  |  |
| PLAUR | UROKINASE | modulator\|inducer |  |  |  |
| EGFR | PANITUMUMAB | suppressor\|antibody\|inhibitor |  |  |  |
